# Bezold’s Abscess: A Case Report and Review of Cases Over 20 Years

**DOI:** 10.7759/cureus.21533

**Published:** 2022-01-23

**Authors:** Abdullah S Alkhaldi, Mohammed Alwabili, Thamer Albilasi, Khabti Almuhanna

**Affiliations:** 1 College of Medicine, King Saud Bin Abdulaziz University for Health Sciences, Riyadh, SAU; 2 Department of Otolaryngology - Head & Neck Surgery, Prince Sultan Military Medical City, Riyadh, SAU

**Keywords:** bezold abscess, suppurative mastoiditis, complications of acute mastoiditis, acute mastoiditis, bezold’s abscess

## Abstract

Bezold’s abscess (BA) is a severe and rare extracranial complication of suppurative acute mastoiditis. The diagnosis of BA requires a high index of suspicion due to its rarity. In this study, we present a rare case of BA, in addition to a review of literature over 20 years. We searched for all cases in English literature from 2000 to 2020 in PubMed and found 27 cases (28 cases including the current case). BA was more prevalent in males (17/28, 60.7%) and adults (17/28, 60.7%). Of the 28 cases, six were associated with cholesteatoma and another six cases occurred with concomitant sinus thrombosis.

## Introduction

Bezold's abscess (BA) is caused by pus draining through the medial wall of the mastoid process and producing a suppurative collection in the digastric sulcus [1]. This abscess is named after Friedrich von Bezold, a German otologist who first reported a neck abscess in the sternocleidomastoid muscle in 1881 [2]. This suppurative collection might track to the digastric muscle and involve the retromaxillary fossa along the occipital artery. If left untreated, further deep extension can occur. In case of a severe infection of the mastoid bone, the suppurative contents of the mastoid air cells may descend along the upper insertion of the sternocleidomastoid muscle, causing pus to accumulate between the muscle and the fascia. The contents of BA can extend to the mediastinum if not treated appropriately and promptly, resulting in acute mediastinitis, which has a 70% fatality rate [3]. Mastoiditis can affect people of all ages, although it is more common in older adults [4]. The discovery of antibiotics has revolutionized the course of mastoiditis and significantly decreased its complications. As a result, BA has become less severe and less frequent. The clinical significance of the mastoid bone is linked to the nearby anatomical structures, such as the middle cranial fossa, posterior cranial fossa, sigmoid and lateral sinuses, facial nerve canal, semicircular canals, and petrous tip of the temporal bone. This study describes a case of BA, in addition to a review of literature from 2000 to 2020.

## Case presentation

A 46-year-old male presented to our emergency department complaining of left otorrhea, high fever, left post-auricular pain, and a painful left neck swelling, which increased in size over the past four days with decreased hearing. The patient had a history of right otorrhea periodically for three years that was treated multiple times with oral antibiotics and local antibiotic ear drops. In addition, the patient had a history of Bell’s palsy three years previously, which resolved with medical treatment and facial exercise. The patient denied a history of vertigo, tinnitus, airway symptoms, dysphagia, odynophagia, or trismus. There was no history of previous ear surgery.

**Figure 1:**
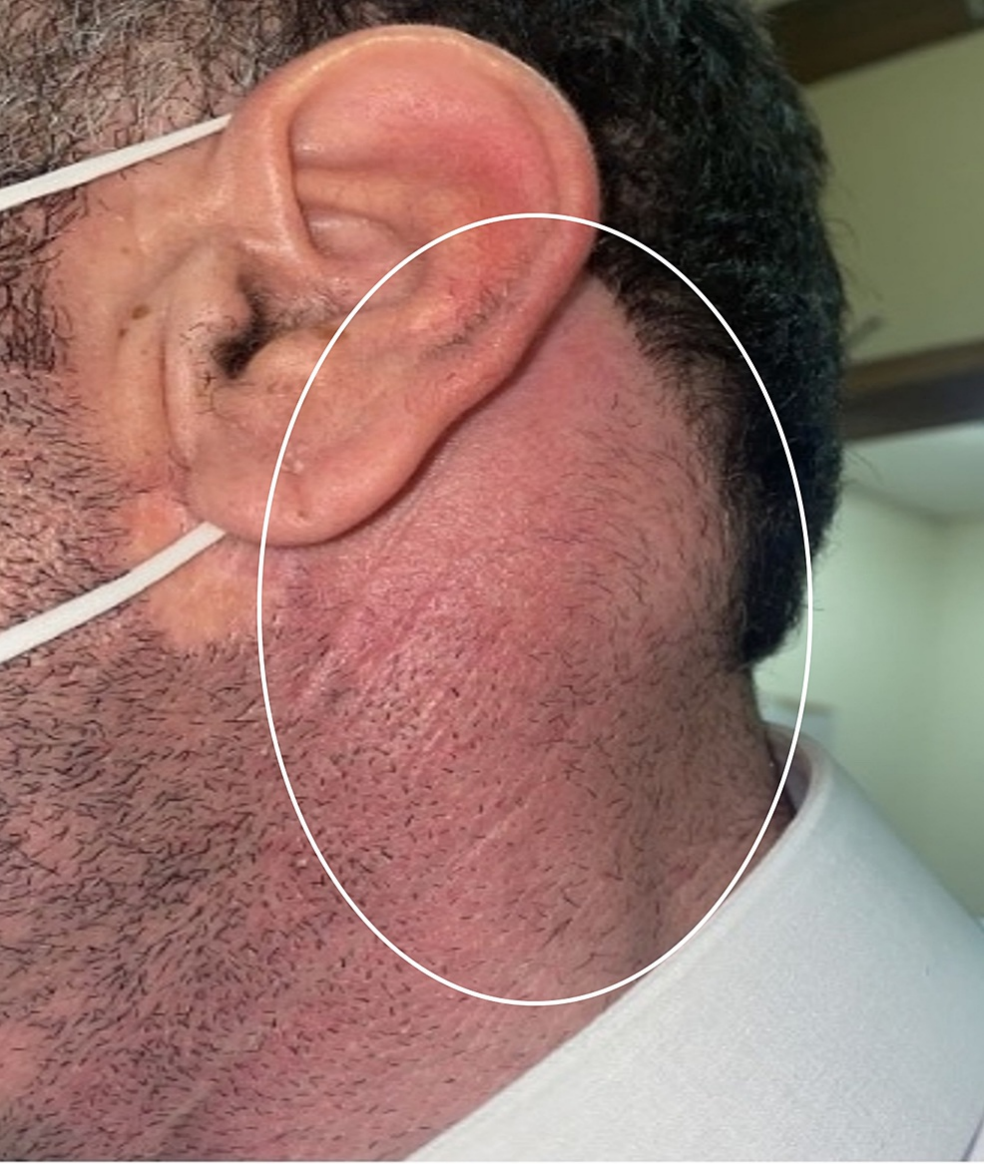
Left lateral diffuse swelling over the mastoid bone extending down to the upper sternocleidomastoid muscle.

On examination, the patient was febrile; he experienced pain, but his vital signs were stable. There was post-auricular erythema and a left lateral diffuse swelling over the mastoid bone extending down to the upper sternocleidomastoid muscle with tenderness and fluctuation (Figure 1). On otoscopy, the right ear was unremarkable. The left ear had a clear external auditory canal and dull tympanic membrane. Rinne test was negative for the left ear but positive for the right ear. Weber test indicated lateralization to the left side. The eye examination was unremarkable (there was no nystagmus), and the fistula test was negative. Facial movement was symmetrical, suggesting that the facial nerve was intact, and the examination of the other cranial nerves was unremarkable. There were no signs of meningeal irritation or palpable lymph nodes. A flexible scope showed a clear nasopharynx, oropharynx, and hypopharynx. Laboratory results showed increased inflammatory markers (Table 1).

**Table 1:** Laboratory results of the patient

Test	Result
White blood cells	12,000/mm^3^ (mainly neutrophils)
C-reactive protein	60 mg/L
Erythrocyte sedimentation rate	80 mm/hour
Blood sugar	12 mmol

Computed tomography of the head and neck with contrast

**Figure 2:**
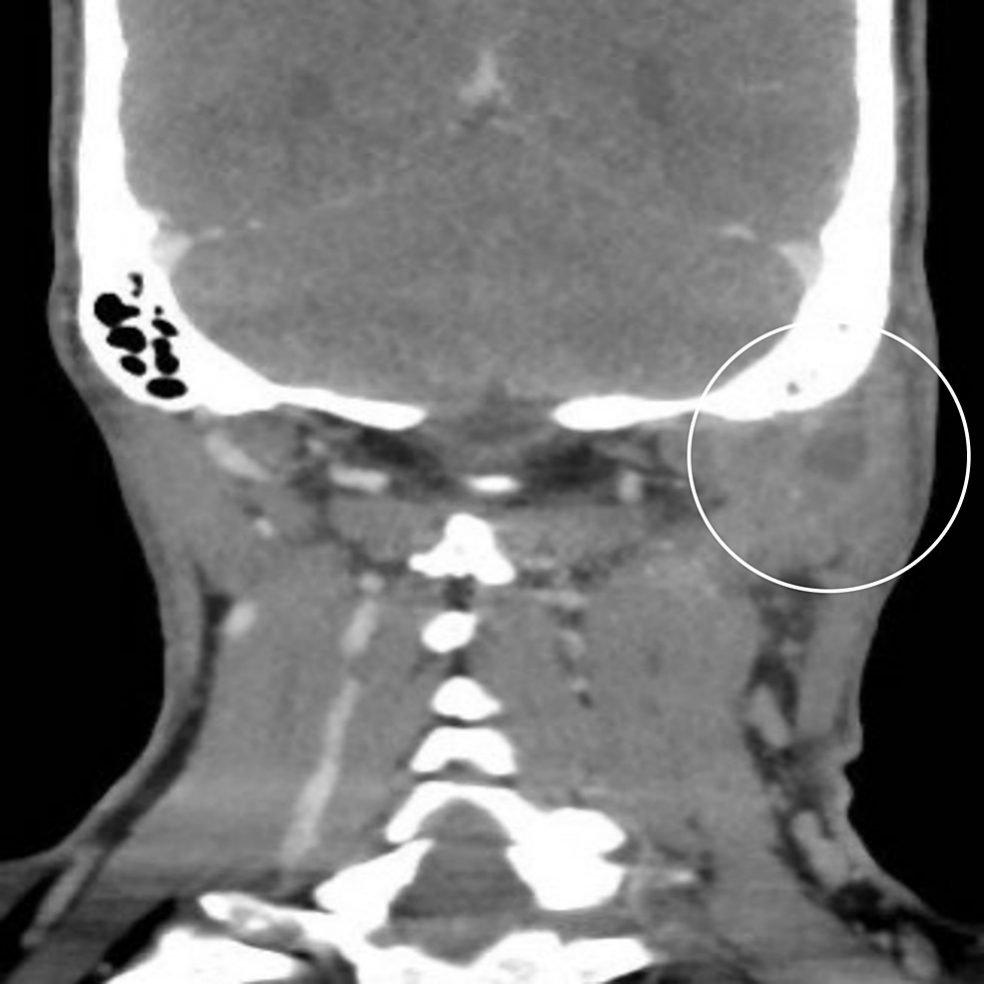
Coronal enhanced CT image of the head and neck showing opacification of the left lateral inferior to the mastoid area with rim enhancement, medial to the sternocleidomastoid muscle, a typical picture commonly seen with Bezold’s abscess.

**Figure 3:**
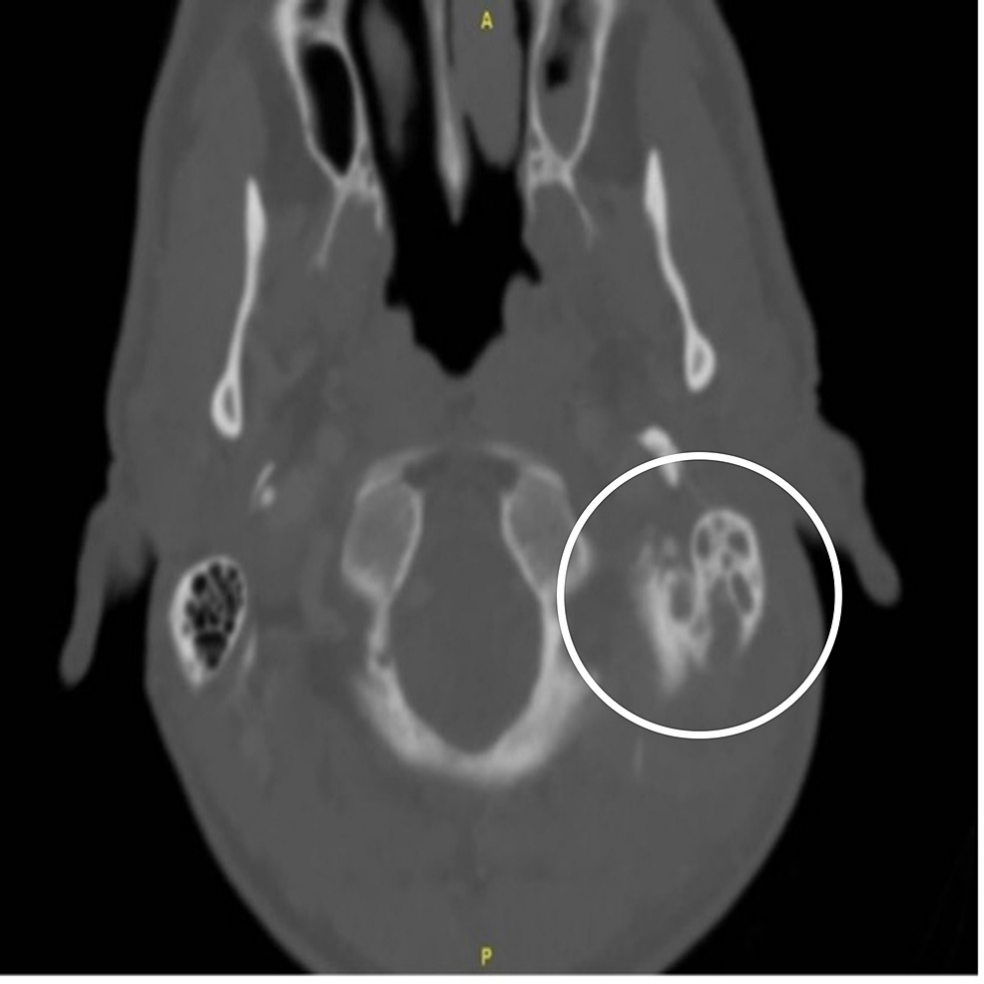
Axial CT at the level of the mastoid showing posterior defect of the left mastoid cortex with complete mastoid opacification.

There was evidence of acute mastoiditis with bony erosive changes in the left mastoid bone and a thick walled peripherally enhancing radiolucency measuring 3.5 x 2.6 x 3.5 cm in the left retrosternal region abutting the sternocleidomastoid muscle posteriorly (Figures 2, 3).

Management

**Figure 4:**
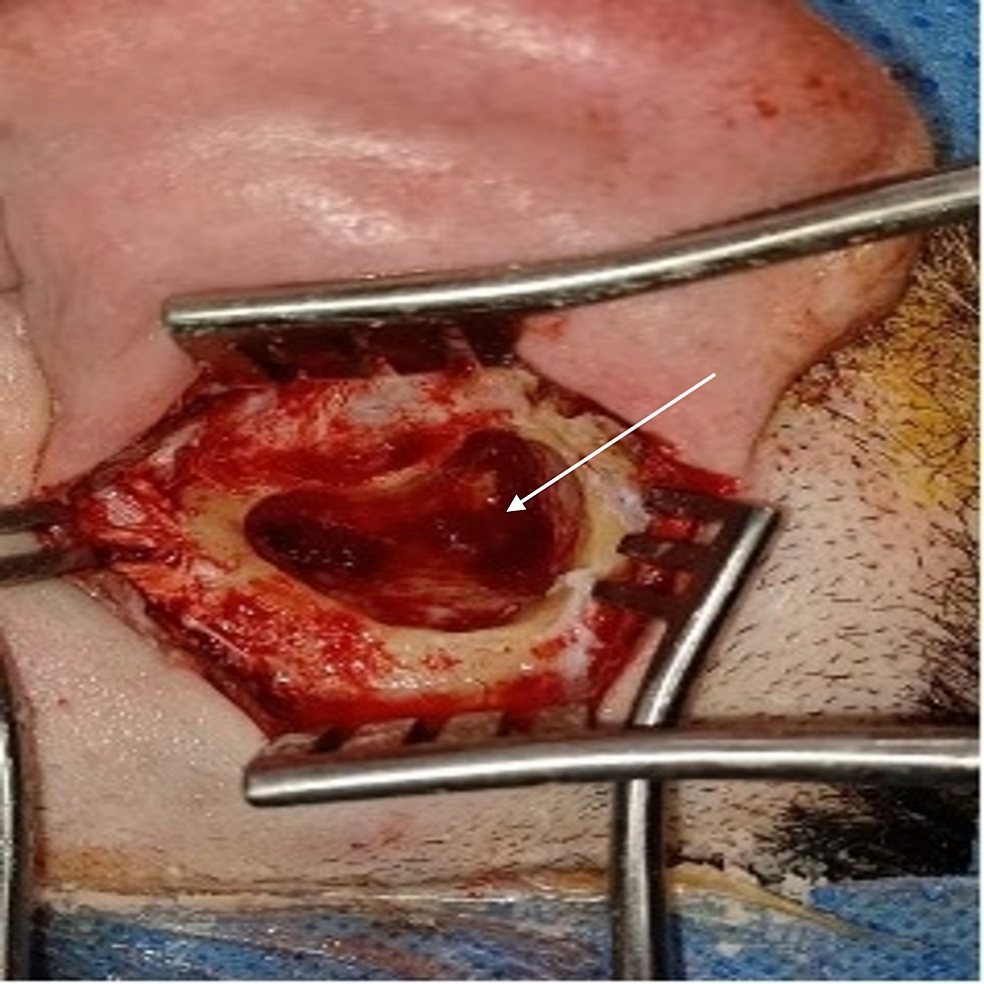
Static view of intra-operative finding showing copious pus collection in the mastoid following cortical mastoidectomy.

On arrival to the emergency department, intravenous (IV) ceftriaxone 2 g and IV paracetamol 1 g were started immediately. On admission, ceftazidime 2 g and metronidazole 500 mg were prescribed. The next day, the patient underwent a cortical mastoidectomy, and an incision and drainage of the abscess under general anesthesia was performed. After the incision, copious pus came out, which was sent for culture (Figure 4). The result showed no growth. Five days after the surgery, the patient was discharged home on ciprofloxacin and clindamycin in a good condition. The patient was followed up in the clinic one week after discharge; he was doing well with no active complaint, and his wound was clean.

## Discussion

BA is a severe and rare extracranial complication of suppurative acute mastoiditis. We searched English literature for all the cases reported from 2000 to 2020 in PubMed. We found 28 cases (including ours), as presented in Table 2. There are other reported cases, which were excluded due to not being in English and not being registered in PubMed. BA as reported in the literature was more common in males (17/28, 60.7%) than in females (11/28, 39.3%). It is also diagnosed more in adults (17/28, 60.7%) than in children 18 years and younger (11/28, 39.3%). The age range was 10 weeks to 77 years. BA is extremely rare in infants and young children due to incomplete mastoid pneumatization [5]. In the absence of complete pneumatization, mastoid bone walls are thick and difficult to erode. Only three cases of BA have been described in patients younger than five years.

**Table 2:** Review of all reported cases of Bezold's abscess in the English Literature between 2000 and 2020 I and D, incision and drainage; IV, intravenous; HIV, human immunodeficiency virus; CSF, cerebrospinal fluid

Case no.	Author	Year of publication	Age	Sex	Management	Culture	Coexistence complications/comorbidities
1	Marioni et al. [5]	2001	18 months	Female	IV cefotaxime	Not available	None
2	Zapanta et al. [6]	2001	17 years	Female	Mastoidectomy + decompression of an epidural abscess + I and D + IV clindamycin, ceftriaxone, and vancomycin + myringotomy and tube	Alpha-hemolytic streptococci	Multiple dural sinus thrombosis
3	Uchida et al. [7]	2002	25 years	Male	Mastoidectomy + I and D + IV antibiotics	Staphylococcus + Veillonella species	Cholesteatoma
4	Jose et al. [8]	2003	19 years	Male	I and D + IV flucloxacillin	Staphylococcus aureus	none
5	Schöndorf et al. [9]	2004	10 weeks	Female	Mastoidectomy + IV antibiotic	No growth	none
6	Ching et al. [10]	2006	14 years	Male	Mastoidectomy + IV ceftriaxone and metronidazole	Streptococcus milleri	Lateral sinus thrombosis, poststreptococcal glomerulonephritis
7	Bhat and Manjunath [11]	2007	12 years	Male	I and D + IV ceftazidime + temporal craniotomy + radical mastoidectomy	Pseudomonas aeruginosa	Pyogenic meningitis + cholesteatoma + sigmoid sinus thrombosis + cerebellar abscess + CSF otorrhea + perilymph fistula
8	McMullan [12]	2009	8 years	Male	Mastoidectomy + insertion of grommet tube + I and D + IV cefotaxime, clindamycin, vancomycin, meropenem	No growth	Sigmoid sinus thrombus
9	Vlastos et al. [13]	2010	3 years	Female	Mastoidectomy + IV clindamycin and ceftriaxone	Streptococcus pneumoniae	Sigmoid sinus thrombosis and occipital osteomyelitis
10	Patel et al. [14]	2010	35 years	Male	Mastoidectomy + I and D + IV piperacillin/tazobactam and vancomycin	Not available	HIV
11	Sheikh et al. [15]	2011	26 years	Male	I and D of neck abscess	acid-fast bacilli	Prior cholesteatoma and mastoidectomy
12	Mascarinas et al. [16]	2011	77 years	Female	Mastoidectomy+ I and D + IV antibiotic	Streptococcus viridans	Postradiation
13	Li and Ren [17]	2012	32 years	Female	Mastoidectomy and I and D	Not available	Cholesteatoma
14	Janardhan et al. [18]	2012	60 years	Male	Mastoidectomy + I and D + IV antibiotics	Not available	Congenital cholesteatoma
15	Secko and Aherne [19]	2013	32 years	Male	I and D + IV ceftriaxone	Not available	HIV
16	Nelson and Jeanmonod [20]	2013	12 years	Female	IV clindamycin + dexamethasone + I and D	Streptococcus pyogenes	None
17	Lionello et al. [21]	2013	35 years	Male	Mastoidectomy + I and D + IV ceftriaxone and metronidazole	No growth	Cholesteatoma
18	Pradhananga [22]	2014	14 years	Female	modified radical mastoidectomy with type III tympanoplasty + I and D + broad spectrum antibiotics	Not available	none
19	Al-Baharna et al. [23]	2016	73 years	Male	Mastoidectomy + I and D + IV ceftriaxone	Peptostreptococcus species	Diabetes, hypertension, renal impairment, and cardiomyopathy
20	Quoraishi et al. [24]	2016	44 years	Male	IV cefotaxime + myringotomy with tube insertion + cortical mastoidectomy + I and D	No growth	none
21	Nasir and Asha'ari [25]	2017	52 years	Male	IV ampicillin/sulbactam + modified radical mastoidectomy + I and D	Klebsiella pneumoniae	Diabetes, left facial nerve palsy grade V
22	Yaita et al. [26]	2018	70 years	Female	IV ampicillin/sulbactam + IV ceftriaxone	Streptococcus constellatus	Parkinson's disease + thrombosis or right transverse sinus + acute infarction of the right cerebellum
23	Eswaran et al. [27]	2019	15 years	Female	topical and systemic antibiotics + modified radical mastoidectomy	Methicillin-sensitive Staphylococcus aureus + acid-fast bacilli	None
24	Katayama et al. [28]	2018	52 years	Male	IV ceftriaxone + metronidazole + drainage	Streptococcus pneumoniae	Diabetes + hyperlipidemia
25	Mustafa et al. [29]	2018	14 years	Female	IV ceftriaxone and vancomycin + cortical mastoidectomy + myringotomy with tube insertion + abscess drainage	Streptococcus pneumoniae	None
26	Malik et al. [30]	2019	55 years	Male	right tympanomastoidectomy + canaloplasty + incision and drainage of Bezold’s abscess + IV vancomycin, cefepime, and metronidazole	Not available	Skull base osteomyelitis
27	Lyoubi et al. [31]	2020	62 years	Male	IV ceftriaxone and moxifloxacin + wide mastoidectomy + surgical drainage of abscess	Not available	None
28	Our case	2021	46 years	Male	IV ceftriaxone + cortical mastoidectomy + I and D of abscess	No growth	None

The diagnosis of BA requires a high index of suspicion due to its rarity. Patients may not show any signs of sepsis, and clinicians should be aware of this complication in patients with otitis media or acute mastoiditis. The most prevalent reported clinical signs and symptoms were fever, otalgia, neck edema, neck pain, otorrhea, torticollis, facial paralysis, and hearing loss [5]. Our patient was mainly complaining of otorrhea, high fever, post-auricular pain, and a painful left neck swelling increasing in size.

The mastoid tip, which is pneumatized in adults, is composed of thin-walled air cells. The lateral wall of the mastoid is composed of thicker bone than that of the medial wall. The lateral wall serves as an insertion site for the sternocleidomastoid, digastric, splenius capitis, and longissimus capitis muscles. Pus in the mastoid erodes through the mastoid tip, the area of least resistance, which is inferior and medial. The abscesses are formed deep in the neck muscles, which delay its detection. Another factor that could contribute to the delayed diagnosis is the unfamiliarity of the disease to the clinician.

BA has been linked to lateral sinus thrombosis [32], which is caused by the compression or thrombosis of the internal jugular vein. We found six cases with concomitant sinus thrombosis. Similarly, six cases were associated with either primary or recurrent cholesteatoma. The presence of cholesteatoma in mastoiditis blocks the middle ear aditus and directs the inflammatory process to the mastoid tip. Patients with a history of cholesteatoma appear to be at an increased risk for BA.

The diagnosis can be challenging because of rarity of this abscess and the variable signs and symptoms. Computed tomography (CT) and magnetic resonance imaging (MRI) images can locate the abscess, as described in Table 3. The CT scan in the current case of BA indicated an ipsilateral opacification of the middle ear and mastoid cavity, often associated with bony erosions. The collection of pus can be detected along the sternocleidomastoid muscle. Contrasted CT images of the temporal bone and neck are most important for both the diagnosis and subsequent surgical treatment. In addition, the laboratory evaluation is often supportive in diagnosis, as the leukocyte count is usually high and the erythrocyte sedimentation rate elevated.

**Table 3:** Radiological findings of reported cases of Bezold's abscess CT, computed tomography; MRI, magnetic resonance imaging

Author	Modality	Findings
Marioni et al. [5]	CT	Non-erosive debris throughout the middle ear cavity and mastoid on the right side and thickening of prevertebral and retropharyngeal spaces on the same side.
Zapanta et al. [6]	CT	Left-sided coalescent mastoiditis and pansinus opacification. Left-side sigmoid sinus showing the filling defect or “empty delta sign”.
Uchida et al. [7]	CT	Round-shaped soft tissue mass from the mastoid process through the sigmoid sinus sulcus causing extensive bony destruction of both the temporal and occipital bones.
Jose et al. [8]	MRI	Opacification of the left middle ear and mastoid air cells, lateral sinus thrombosis, and adjacent area of meningeal inflammation.
Schöndorf et al. [9]	MRI	Bright signal shows inflammation in mastoid bone with involvement of insertion of the left sternocleidomastoid muscle.
Ching et al. [10]	CT	Opaque left mastoid air cell as well as a filling defect in the left sigmoid sinus in keeping with septic thrombophlebitis. Note also the small extradural collection adjacent to the sigmoid sinus. More inferiorly, there is a septated Bezold’s abscess with an enhancing rim just posterior to the tip of the left mastoid process.
Bhat and Manjunath [11]	CT	Erosion of the sinus plate, the cerebellar abscess, and the dilation of the ventricles.
McMullan [12]	CT	Bilateral otomastoiditis with associated bony destruction in the mastoid cavities, extension into the right sigmoid sinus, and extension into the neck inferiorly, consistent with an abscess related to the deep surface of the right sternocleidomastoid muscle. There was also partially occlusive thrombus in the right sigmoid sinus.
Vlastos et al. [13]	CT	Osteolytic process within the left mastoid, edema, and a small abscess formation in the left upper neck region (Bezold’s abscess) and thrombosis of the ipsilateral sigmoid sinus.
Patel et al. [14]	CT	Left-sided coalescent mastoiditis. A 3.6 x 1.8 cm abscess at the level of the left mastoid tip tracked deep to the left sternocleidomastoid and extended medially and anteriorly into the pre-vertebral space surrounding the anterior arch of C1. Contrast study showed a hypoplastic left jugular bulb and no flow in the internal jugular bulb or sigmoid sinus.
Sheikh et al. [15]		Not available.
Mascarinas et al. [16]	CT	On the left side, coalescent mastoiditis and an abscess within the superior aspect of the SCM muscle communicating with the mastoid cavity through a bony dehiscence of the mastoid tip, consistent with a Bezold’s abscess.
Li and Ren [17]	MRI	Two masses located in the right mastoid cavity and neck measuring 4.0 cm x 3.0 cm and 5.0 cm x 3.0 cm in diameter. The masses had smooth, well-defined outlines with intermediate signal intensity on T1-weighted and hyperintense on T2-weighted without post-contrast enhancement.
Janardhan et al. [18]	CT	Soft tissue attenuation of the right mastoid antrum with absence of air cells. There was an absence of medial wall of mastoid antrum in the region of Trautmann’s triangle and sinus plate. Also, a bony deficiency was seen in the posterior meatal wall.
Secko and Aherne [19]	CT	Edema of the maxillary, sphenoid, and mastoid air cells consistent with sinusitis. CT of the neck at the level of the mandible demonstrated a small hypodense lesion in the area of the sternocleidomastoid muscle consistent with abscess.
Nelson and Jeanmonod [20]	CT	Extensive opacification of the left mastoid temporal bone consistent with acute otomastoiditis. In addition, there was bony erosion and destruction of the mastoid tip inferiorly with extensive surrounding inflammation within the adjacent soft tissues and a 1 cm peripherally enhancing, developing Bezold’s abscess with diffuse reactive adenopathy within the left neck.
Lionello et al. [21]	CT	At the C1-C2 level, there was soft tissue edema on the left side involving the sternocleidomastoid muscle, which appeared swollen and unevenly enhanced; focal areas of necrosis.
Pradhananga [22]	CT	Air fluid collection in the left mastoid and middle ear cavity. There was erosion of the mastoid cavity and sinus plate. A defect was noted in the medial wall of the left mastoid cavity. Fluid collection with air foci within was also noted in soft tissue adjacent to the left mastoid cavity and extending into the neck, suggestive of abscess.
Al-Baharna et al. [23]	MRI	Abscess collection within the sternocleidomastoid muscle continuous with mastoid collection.
Quoraishi et al. [24]	CT	Right mastoiditis with bony erosion anterior to the sigmoid venous sinus. Overlying superficial abscess, tracking inferiorly deep to the sternocleidomastoid muscle, and anteriorly to the margin of the styloid process and carotid sheath.
Nasir and Asha'ari [25]	CT	Soft tissue density within the left middle ear cavity and mastoid air cells with wide erosion at the posteroinferior part of the mastoid, medial to the mastoid tip. There was abscess collection deep to the sternomastoid muscle below the mastoid tip erosion. The collection extended inferiorly along the paravertebral muscles until the seventh cervical vertebrae.
Yaita et al. [26]	CT	Polycystic lesions (abscesses) on her right posterior and on the lateral region of her neck, thrombosis in her right internal jugular vein, and multiple nodules on her bilateral lung fields (septic emboli) were present.
Eswaran et al. [27]	CT	Soft tissue opacification in right middle ear cavity and mastoid antrum with breach in the mastoid tip. Breach in the continuity of the right mastoid bone was also seen posteriorly abutting the right sigmoid sinus. There was erosion in the posterior bony canal wall. Hypodense area with thick enhancing wall was noted medial to the superior part of the sternocleidomastoid muscle, suggestive Bezold’s abscess.
Katayama et al. [28]	CT	Revealed multiple abscesses spread from the right temporal bone to the right sternocleidomastoid muscle. It also demonstrated osteolysis at his right mastoid process.
Mustafa et al. [29]	CT	Irregular hypodensity below the right mastoid and right half of the occipital bone surrounded with postcontrast (red circle) increase of density represent the abscess formation.
Malik et al. [30]	CT	Right-sided chronic mastoiditis, erosion of the inferior mastoid cells, extension of the infection into the neck spaces, and formation of a Bezold’s abscess in the ipsilateral sternocleidomastoid muscle, extending into the retropharyngeal space
Lyoubi et al. [31]	CT	Right-sided chronic mastoiditis, erosion of the inferior mastoid cells, and cervical cellulitis collected in the right sternocleidomastoid muscle measuring 33 × 15 mm extended to 40 mm.

Mastoiditis has a similar bacteriology to acute otitis media, with *Streptococci* species the major pathogens. In our review, *Streptococci* were the most frequent causative organisms. However, multiple organisms, both gram positive and negative, as well as anaerobes, were cultured. Antibiotics effective against gram-positive organisms should be initiated since they are the most frequent causative pathogens. Subsequently, culture-based antibiotics can be described. In addition to the IV antibiotics, a surgical intervention (mastoidectomy and abscess drainage) is required for the effective management and prevention of further complications. Of the 28 patients with data regarding treatment, 21 (75%) underwent a mastoidectomy (Table 2). The other eight cases required no mastoidectomy (two were children without a fully pneumatized mastoid bone). This suggests that the surgical treatment can be tailored to mastoid bone pneumatization and the neck abscess extension.

## Conclusions

BA is a severe and rare extracranial complication of suppurative acute mastoiditis. The diagnosis of BA requires a high index of suspicion due to its rarity and the variable signs and symptoms. In this study, we presented a rare case of BA that was managed by IV antibiotics, incision and drainage of the abscess, and cortical mastoidectomy. In addition, we presented a review of BA cases over 20 years (2000-2020). In almost all the cases, the gold standard management was IV antibiotics, drainage of the abscess, and mastoidectomy.
